# Coronavirus Nsp1: Immune Response Suppression and Protein Expression Inhibition

**DOI:** 10.3389/fmicb.2021.752214

**Published:** 2021-09-28

**Authors:** Shuai Yuan, Shravani Balaji, Ivan B. Lomakin, Yong Xiong

**Affiliations:** ^1^Department of Molecular Biophysics and Biochemistry, Yale University, New Haven, CT, United States; ^2^Department of Dermatology, Yale University School of Medicine, New Haven, CT, United States

**Keywords:** coronavirus, nonstructural protein 1, immune suppression, translation inhibition, vaccine

## Abstract

Coronaviruses have brought severe challenges to public health all over the world in the past 20years. SARS-CoV-2, the causative agent of the COVID-19 pandemic that has led to millions of deaths, belongs to the genus beta-coronavirus. Alpha- and beta-coronaviruses encode a unique protein, nonstructural protein 1 (Nsp1) that both suppresses host immune responses and reduces global gene expression levels in the host cells. As a key pathogenicity factor of coronaviruses, Nsp1 redirects the host translation machinery to increase synthesis of viral proteins. Through multiple mechanisms, coronaviruses impede host protein expression through Nsp1, while escaping inhibition to allow the translation of viral RNA. In this review, we discuss current data about suppression of the immune responses and inhibition of protein synthesis induced by coronavirus Nsp1, as well as the prospect of live-attenuated vaccine development with virulence-attenuated viruses with mutations in Nsp1.

## Introduction

Three highly pathogenic human coronaviruses (hCoVs) have emerged in the last two decades, including severe acute respiratory syndrome coronavirus (SARS-CoV; Ksiazek et al., 2003; Rota et al., 2003), Middle East respiratory syndrome coronavirus (MERS-CoV; Bermingham et al., 2012; Zaki et al., 2012), and severe acute respiratory syndrome coronavirus 2 (SARS-CoV-2), which caused the worldwide pandemic COVID-19 since late 2019 (Coronaviridae Study Group of the International Committee on Taxonomy of Viruses, 2020). Coronaviruses are members of the family *Coronaviridae* under the order *Nidovirales* and are among the largest RNA viruses containing a positive-sense single-stranded RNA genome (Masters, 2006; Lim et al., 2016; Lefkowitz et al., 2018). Coronaviruses are further subgrouped into four genera: alpha-coronavirus (alpha-CoV), beta-coronavirus (beta-CoV), gamma-coronavirus (gamma-CoV), and delta-coronavirus (delta-CoV; Gorbalenya et al., 2004; McBride and Fielding, 2012; Lefkowitz et al., 2018). Of these four genera, hCoVs are associated with both alpha- and beta-CoVs and can cause symptoms ranging from mild disease to severe respiratory tract illness (Snijder et al., 2003; Woo et al., 2012). The alpha-CoV strains HCoV-229E and HCoV-NL63 have mostly shown to cause symptoms of common cold, whereas the beta-CoV strains SARS-CoV, MERS-CoV, and SARS-CoV-2 have been characterized by severe illness, high infectivity, and a substantial fatality rate (Sizun et al., 1995; Gagneur et al., 2002; Vabret et al., 2009; V’kovski et al., 2021).

The genome of hCoVs shares a common structure: The first two-thirds of the viral RNA contain two overlapping reading frames ORF1a and ORF1b that encode large polyproteins, which are then cleaved into 16 mature nonstructural proteins (Nsps) that play important roles in viral replication and spread. The remaining one-third of the genome encodes for the structural and accessory proteins [spike (S), envelope (E), membrane (M), nucleocapsid (N), etc.] that are critical for the structural elements of progeny viruses. The genome also contains 5' and 3' UTRs that fold into conserved higher-order structures and have functional roles in viral replication (Snijder et al., 2003). When the viral genome is released into the host cell cytoplasm, the 16 Nsps are among the first viral proteins to be expressed in the cell. These Nsps enable viral replication through both direct and indirect mechanisms. Among them is nonstructural protein 1 (Nsp1), a protein unique to alpha-CoVs and beta-CoVs. Nsp1 is the product of the N terminus of the first open-reading frame ORF1a and serves to suppress host gene expression and the host immune response. Nsp1 plays key roles in the viral lifecycle. It is reported that an in-frame (aa 79–89) deletion in Nsp1 of SARS-CoV-2 correlates with lower viral load (Lin et al., 2021). Here we will discuss the immune response suppression and translation inhibition induced by Nsp1 of coronaviruses, mainly focusing on the beta-CoVs.

## Nsp1 and the Immune Response Suppression

**Figure 1:**
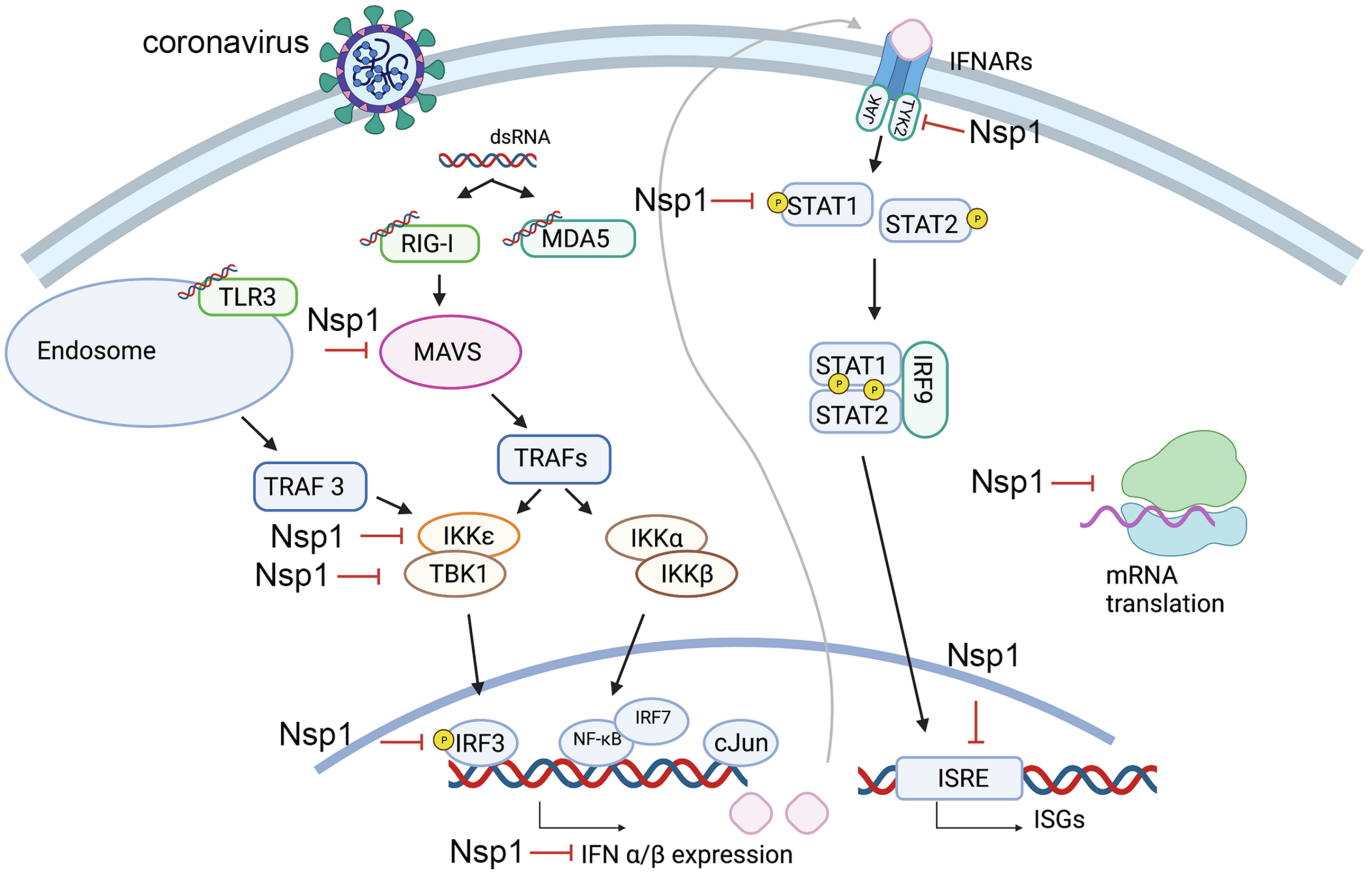
Steps of the innate immune response pathways that potentially be suppressed by nonstructural protein 1 (Nsp1). Key steps that are found inhibited by Nsp1 are labeled.

One of the main determinants of a viral infection efficiency is the virus’ ability to evade and suppress the host innate immune responses. The Nsp1 proteins of both alpha-CoVs and beta-CoVs are likely a part of larger coordinated strategies for immune suppression and have been shown to interfere with multiple steps of the immune response pathway (Wang et al., 2010; Shen et al., 2020; Thoms et al., 2020; Figure 1). Though the inhibition of gene expression is global (Kamitani et al., 2006; Narayanan et al., 2008; Yuan et al., 2020), inhibition of antiviral genes is a major contributor to a weakened immune response upon coronavirus infection. Highly studied players of the innate immune system include Type I interferons, such as IFN-α and IFN-β (Isaacs and Lindenmann, 1957; Biron, 1998; Akira et al., 2006). Multiple studies have shown interferon suppression by Nsp1 in both virus-infected and plasmid-transfected (HEK 293T) cell lines (Kamitani et al., 2006; Narayanan et al., 2008; Thoms et al., 2020). The processes involving Type I interferons begin with the recognition of viral signatures, known as pathogen-associated molecular patterns (PAMPs), by pathogen recognition receptors (PRRs; Medzhitov and Janeway, 1997; De Maeyer and De Maeyer-Guignard, 1998). The most common signature in the coronavirus lifecycle is viral double-stranded RNA (dsRNA) produced during replication, which can be recognized by the retinoic acid-inducible gene I (RIG-I), melanoma differentiation antigen 5 (MDA5), and RNA-activated protein kinase (PKR) in the cytoplasm, as well as toll-like receptor-3 (TLR3) in endosomes (Alexopoulou et al., 2001; Yu and Levine, 2011; Kindler and Thiel, 2014; Weber et al., 2016). Depending on the PRR, multiple sets of partially cross-talking immune signaling cascades are triggered to initiate transcription of type I interferons (Medzhitov and Janeway, 1997). For example, the recruitment domain of the RIG-I and MDA5, collectively termed RIG-I like receptors (RLRs; Loo and Gale, 2011), interact with the mitochondrial antiviral adaptor protein (MAVS), which converges on multiple TNF receptor-associated factors (TRAFs; Yoneyama and Fujita, 2009; Fang et al., 2017). Various TRAFS activate the kinases TBK1 (TANK-binding kinase 1) and IKKε (inhibitor of NF-κB kinase epsilon) that phosphorylate transcription factors including IRF3 (IFN regulatory factors), which can form homo- or heterodimers. Dimerization of these transcription factors allows these molecules to translocate to the nucleus to initiate gene expression of IFN-α and IFN-β and other immunoregulatory cytokines (Medzhitov and Janeway, 1997; Biron, 1998; De Maeyer and De Maeyer-Guignard, 1998; Kindler et al., 2016). The endosomal pathway stemming from TLR3 can similarly activate IRF3 through another TRAF (TRAF3; Alexopoulou et al., 2001; O’Neill et al., 2013). Simultaneously, MAVs can trigger alternate TRAFS to recruit IKKα and IKKβ to remove repression of the transcription factors NF-κB, which is also involved in IFN and interleukin gene expression (IL-6, IL-8; Totura and Baric, 2012). Upon expression of type I interferons, these molecules are secreted and bind to cognate IFN membrane receptors (IFNARs) that activate Janus kinase 1 (JAK1) and Tyrosine kinase 2 (TYK2). These kinases phosphorylate signal transcription proteins STAT1 and STAT2, which form a heterodimer and associate with the regulatory factor IRF9 to form the IFN-stimulated gene (ISG) factor 3. This complex is translocated to the nucleus where it recognizes IFN-I-stimulated response elements (ISREs), activating an amplified expression of hundreds of ISGs that allow for an antiviral response (Samuel, 2001; Schneider and Sari, 2014; Kindler et al., 2016; Xia et al., 2020).

It has been observed that different strains of hCoVs inhibit IFN-I with varying efficiencies. Xia et al. (2020) designed an rLuc-replicon of nearly the entire SARS-CoV-2 genome and created chimeras by replacing the SARS-CoV-2 Nsp1 with that of SARS-CoV. Nsp1 of SARS-CoV-2 was shown to be more efficient in suppressing STAT1 and STAT2 phosphorylation than Nsp1 of SARS-CoV or MERS-CoV, suggesting that SARS-CoV-2 Nsp1 is more efficient at IFN-I suppression by blocking STAT1 and STAT2 phosphorylation. In a subsequent screening experiment to identify at which precursor step IFN production is inhibited, the cloned gene of SARS-CoV-2 Nsp1 was cotransfected into HEK293T cells with a plasmid expressing distinct components in the RIG-I pathway coupled to a luciferase reporter. Additionally, IRF3/5D, a phosphor-mimic of activated IRF3, was also included in the screen to assess for rescue of pathway suppression upstream of this process. It was found that luciferase activity was clearly lowered when Nsp1 was coexpressed with either MAVS, TBK1, or IKKε; however, this suppression was not substantially rescued with IRF3/5D, as seen in the cases of other SARS-CoV-2 proteins. These results suggest that inhibition of IFN production by Nsp1 may be both upstream and downstream of IRF3 (Xia et al., 2020). It was also reported that Nsp1 of SARS-CoV directly targets IRF3 phosphorylation and localization (Kamitani et al., 2006). For events downstream of IRF3, multiple studies have confirmed that Nsp1 inhibits STAT1 phosphorylation and nuclear translocation without affecting STAT1 expression or phosphorylation of STAT2, JAK1, and TYK2 (Wathelet et al., 2007; Xia et al., 2020). This phenomenon is most likely conserved in alpha-CoVs and beta-CoVs, as inhibition of STAT-1 phosphorylation at S727 has also been observed in strains of SARS-CoV and the alpha-CoVs HCoV-229E and HCoV-NL63 (Shen et al., 2020). Recent studies suggest that in addition to affecting STAT1 phosphorylation, Nsp1 of SARS-CoV-2 may also deplete levels of both TYK2 and STAT2, providing another mechanism by which Nsp1 may inhibit the host immune response (Kumar et al., 2021a).

In a separate study to assess the immune response effects of SARS-CoV Nsp1, IFN-β expression was assayed through similar methods of Nsp1 transfection into HEK293T cells, assessing activity from a reporter plasmid encoding an IFN transcription factor (Wathelet et al., 2007). Results suggested that Nsp1 is able to inhibit activation of NF-κB, IRF3, IRF7, and the transcription factor c-Jun. Accordingly, Nsp1 expression was also observed to inhibit the expression of ISG proteins (ISG15 and ISG56; Wathelet et al., 2007).

## Nsp1 Structure

**Figure 2:**
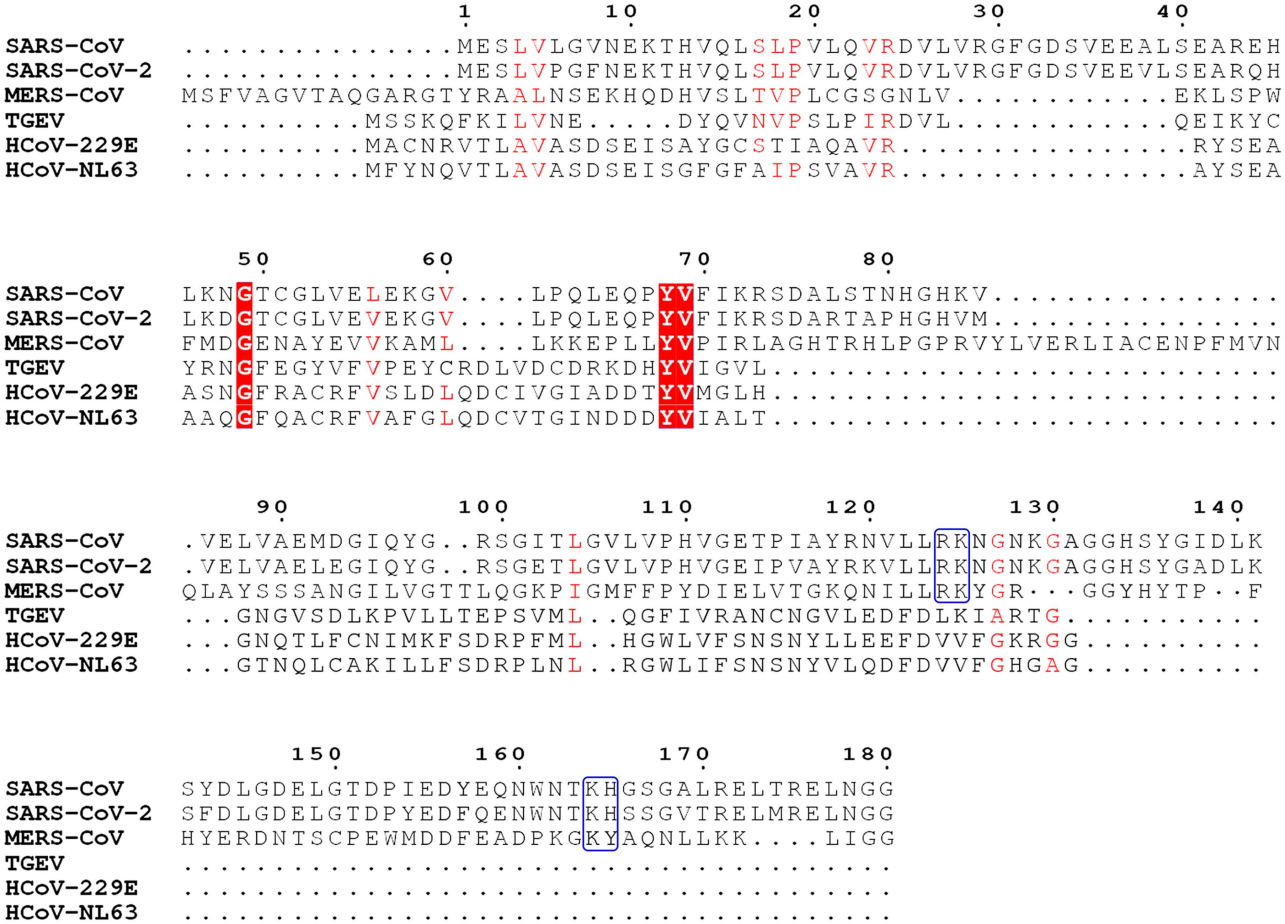
Sequence analysis of Nsp1 from severe acute respiratory syndrome coronavirus (SARS-CoV), severe acute respiratory syndrome coronavirus 2 (SARS-CoV-2), Middle East respiratory syndrome coronavirus (MERS-CoV), transmissible gastroenteritis virus (TGEV), HCoV-229E, and HCoV-NL63. Key conserved residues in SARS-CoV, SARS-CoV-2, and MERS-CoV are labeled with the blue boxes. Conserved residues are labeled in red boxes and shades.

**Figure 3:**
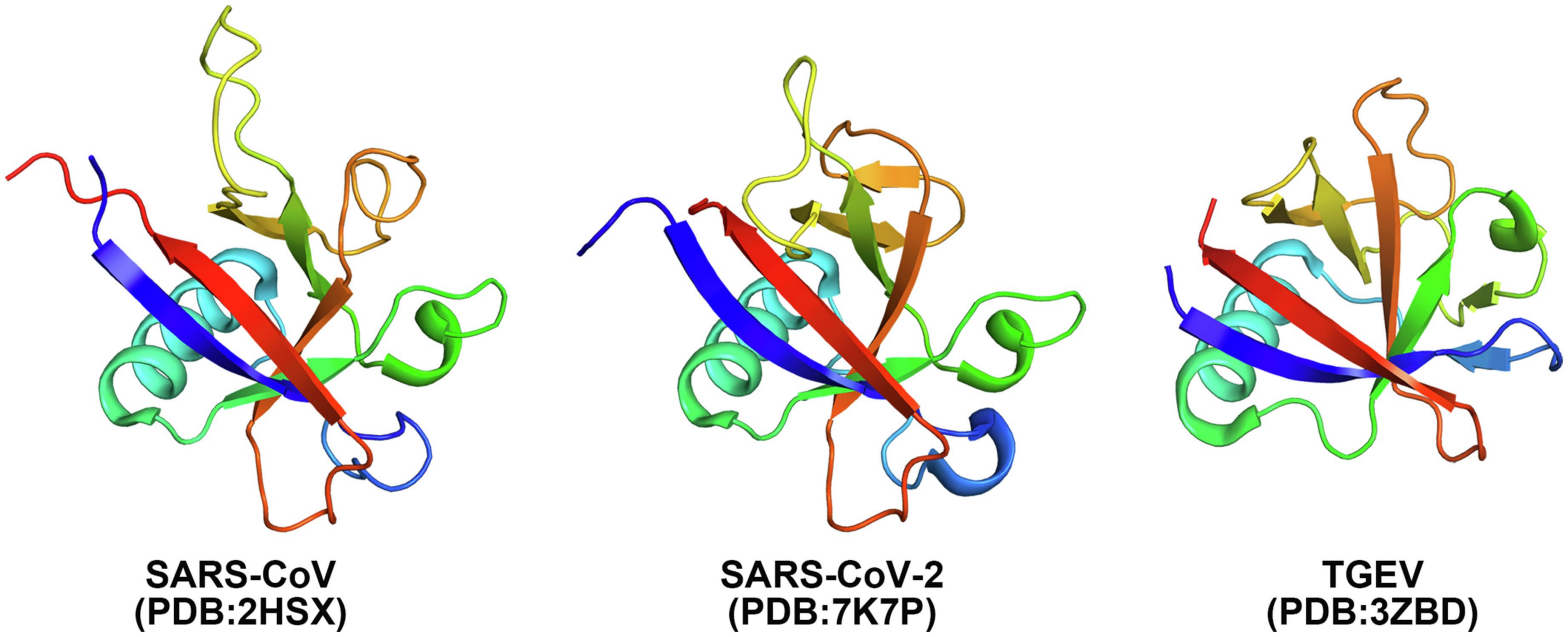
Crystal structures of Nsp1 of SARS-CoV (PDB: 2HSX), SARS-CoV-2 (PDB: 7K7P), and TGEV (PDB: 3ZBD) shown in cartoon. Rainbow coloring from blue to red indicates the N- to C-terminal positions of the residues in the model.

The Nsp1 protein of alpha- and beta-CoVs varies in size within species and genera. Nsp1 proteins encoded by beta-CoVs are about 180aa long, characterized by flexible N- and C-terminal tails and a unique hydrophobic β-barrel motif located in the core domain of the protein, whose structure has been determined for SARS-CoV and SARS-CoV-2 through nuclear magnetic resonance (NMR) analysis or crystallization (Figures 2, 3; Almeida et al., 2007; Clark et al., 2021; Semper et al., 2021). The core structure of SARS-CoV Nsp1 is composed of an antiparallel β-barrel capped by an α-helix, two parallel 3_10_ helices and an additional β-strand (Almeida et al., 2007). SARS-CoV-2 Nsp1 shares ~91% similarity in protein sequence with SARS-CoV Nsp1. The overall structure of the SARS-CoV-2 Nsp1 core domain is similar to that of SARS-CoV (Figure 3; Clark et al., 2021; Semper et al., 2021). However, there are still marked differences, such as additional 3_10_ helix and β-strand that likely contribute to the protein’s overall stability. Most of these differences occur in solvent-exposed regions, supporting the idea that Nsp1’s hydrophobic core is conserved (Semper et al., 2021). The structure of MERS-CoV Nsp1 has not been experimentally determined, but homology models suggest that a similar hydrophobic core is retained and that the structural conservation is even higher than the sequence identity implies (Semper et al., 2021).

In the case of alpha-CoVs, the Nsp1 protein encoded is much smaller (~110aa) and has a much shorter C-terminal tail when compared to the Nsp1 proteins of beta-CoVs (Figure 2). Although alpha-CoV strains are not recognized as sequence homologs of beta-CoVs, manual sequence alignment and analysis suggest their core structures fold similarly (Shen et al., 2019; Figure 3). The nearly full-length Nsp1 of an alpha-CoV, transmissible gastroenteritis virus (TGEV), has been crystallized and extensive analysis has been conducted to compare the folds of TGEV Nsp1 and SARS-CoV Nsp1 (Jansson, 2013). The structures indeed share the characteristic six-stranded β-barrel motif, though the location of the β-barrel and the adjacent α-helix of TGEV Nsp1 is positioned outward and farther away from each other compared with SARS-CoV Nsp1 structure (Figure 3). Nsp1 models of the HCoV-229E and HCoV-NL63 strains have been predicted and show structural conservation with the SARS-CoV Nsp1 core domain (Wang et al., 2010). Namely, the hydrophobic residues that are critical for the β-barrel motif are conserved or replaced by other hydrophobic residues.

## Nsp1 and Translation Inhibition

Viruses require host cell machineries to replicate and spread. In the viral replication process, host cell translation machinery, such as ribosomes and related components are hijacked by viruses to increase synthesis of viral proteins for replication. In the coronavirus lifecycle, Nsp1 is among the first viral proteins to be expressed and serves to suppress synthesis of host proteins, while allowing viral mRNA to escape this suppression and be translated (Ziebuhr, 2005). Nsp1s of both alpha-CoVs (such as HCoV-229E and HCoV-NL63) and beta-CoVs (such as SARS-CoV and MERS-CoV) suppress the host protein synthesis through multi-pronged strategies that include inhibiting the protein production and inducing degradation of host mRNA. However, this multiplex strategy is not always observed; for example, Nsp1 of TGEV inhibits host protein translation without inducing cleavage of the host mRNA (Table 1; Kamitani et al., 2006, 2009; Narayanan et al., 2008; Wang et al., 2010; Huang et al., 2011; Lokugamage et al., 2015; Terada et al., 2017). It has been observed that the SARS-CoV Nsp1 binds the small ribosomal subunit (40S) directly during translation initiation and inhibits translation of both endogenous mRNA and exogenous genes, including those, whose translation is initiated by the viral internal ribosome entry site (IRES; Kamitani et al., 2009). The viral IRES is a highly structured transcript that allows for translation initiation in cap-independent manner, as seen with both the hepatitis C virus (HCV) IRES and cricket paralysis virus (CrPV) IRES. These two IRESes bind to the 40S subunit directly and the CrPV IRES induces the latter steps of translation without the need for additional translation initiation factors (Wilson et al., 2000; Walsh and Mohr, 2011). SARS-CoV Nsp1 is reported to be able to bind to the 40S subunit together with the HCV IRES but abolishes the binding of the CrPV IRES to the 40S subunit (Lokugamage et al., 2012). Interestingly, SARS-CoV-2 Nsp1, which shares about 91% similarity in protein sequence with SARS-CoV Nsp1, binds to 40S subunit together with CrPV IRES and forms a stable ternary complex (Yuan et al., 2020). Importantly, SARS-CoV Nsp1 also inhibits the formation of the active 80S ribosome by preventing the association of the large ribosomal subunit (60S) with the 48S preinitiation complex and this inhibition is mRNA template-dependent (Kamitani et al., 2009; Lokugamage et al., 2012).

**Table 1:** Summary of coronavirus Nsp1 functions.

	Strain	Translation inhibition	Binding to 40S	mRNA degradation
Alpha-CoV	HCoV-229E	Yes	Yes	Yes
	HCoV-NL63	Yes	Yes	Yes
	TGEV	Yes	No	No
Beta-CoV	SARS-CoV	Yes	Yes	Yes
	MERS-CoV	Yes	No	Yes
	SARS-CoV-2	Yes	Yes	Yes

This phenomenon of ribosome binding by Nsp1 is not only observed in beta-CoVs, but also in alpha-CoVs such as HCoV-229E and HCoV-NL63. The Nsp1 proteins of these two strains have been observed to bind the small ribosomal subunit in the 293T cell extracts (Wang et al., 2010; Shen et al., 2018). Intriguingly, Nsp1 proteins from TGEV and MERS-CoV do not bind the 40S subunit, but can still inhibit host protein translation (Huang et al., 2011; Lokugamage et al., 2015). In TGEV Nsp1, there are two conserved electrostatic patches that may play key roles in TGEV Nsp1’s function to inhibit translation. It is reported that TGEV Nsp1 inhibits translation in cell-free HeLa extracts but not rabbit reticulocyte lysate (RRL), suggesting that a host factor that exists in the former, but not the latter, is needed for TGEV Nsp1 function (Huang et al., 2011). The detailed mechanism of translation suppression by TGEV Nsp1 is still unknown and requires further investigation.

**Figure 4:**
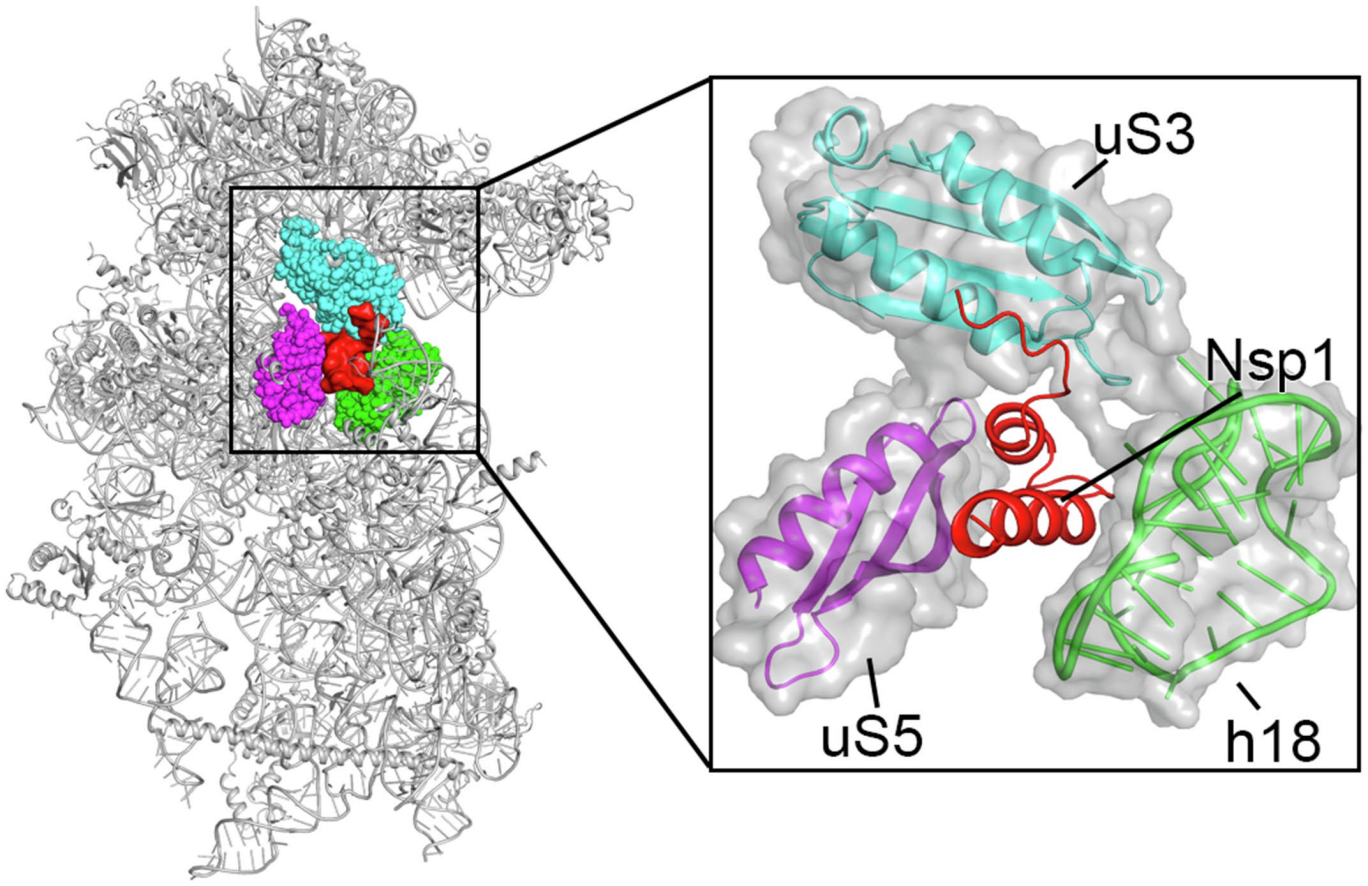
Cryo-EM structure of the Nsp1 C-terminal region-40S ribosomal subunit complex, with Nsp1 (red surface) and the surrounding protein uS3 (cyan sphere representation), uS5 (magenta) and rRNA h18 (green) highlighted. The inset shows zoomed-in view of Nsp1 in cartoon, with the surrounding 40S components in cartoon and surface to illustrate the mRNA entry channel.

Key regions and residues of coronavirus Nsp1 are found to play important roles in host translation inhibition. It has been found that SARS-CoV Nsp1 with a small region deletion (aa 163–170) loses the ability to suppress protein translation (Narayanan et al., 2008). Two key residues, K164 and H165, have been identified as critical for the 40S subunit binding; mutations K164A/H165A in this region have been observed to abolish both binding with the 40S subunit and subsequent inhibition of host protein synthesis (Kamitani et al., 2009). K164 and H165 belong to the C-terminal domain of Nsp1, not present in alpha-CoVs, and are conserved in beta-CoVs (Figure 2). Interestingly, expression levels of the mutants K164A/H165A at 30h post-transfection were much higher than that of the Nsp1-wt, suggesting that Nsp1, but not its C-terminal mutants, also suppressed its own gene expression (Kamitani et al., 2006; Narayanan et al., 2008). Similar results were obtained in the studies of group 2 bat coronavirus Nsp1 (Tohya et al., 2009). In the case of MERS-CoV Nsp1, K181 is the analog of SARS-CoV K164, and it is critical to the protein’s translation inhibition function (Narayanan et al., 2008; Nakagawa et al., 2018; Figure 2). However, this set of residues is not the only feature critical to Nsp1 function. Additionally, Y154A/F157A and R171E/R175E mutations in SARS-CoV-2 Nsp1 have been found to abolish protein translation inhibition in a cell free system (Schubert et al., 2020). These key residues of Nsp1 are critical for interaction with the 40S ribosomal subunit, which indicates the important roles of the C-terminal region of Nsp1 (Schubert et al., 2020; Thoms et al., 2020; Yuan et al., 2020; Figure 4).

In addition to binding to the small ribosomal subunit to suppress protein synthesis, coronavirus Nsp1 also induces host mRNA cleavage, while maintaining the coronavirus RNA. Nsp1 also leads to degradation of some viral RNAs depending on the types of IRES elements. SARS-CoV Nsp1 induces RNA cleavage within the 5'-UTR of capped mRNAs and within the picornavirus type I and type II IRES elements (such as EMCV IRES), while type III (HCV IRES) and type IV (CrPV IRES) are resistant to the Nsp1-induced RNA cleavage (Kamitani et al., 2009). It is shown that SARS-CoV Nsp1 induces the RNA cleavage when Nsp1 is in complex with the 40S subunit and translation initiation factors are loading the mRNA, indicating that some translation initiation factors may be involved in the RNA cleavage (Kamitani et al., 2009). One reason the HCV IRES and the CrPV IRES are not degraded may be that they can bind to the 40S subunit directly without translation initiation factors and subsequent mRNA scanning is not needed for translation initiation. That may suggest that mRNA degradation occurs during mRNA movement through the 40S subunit. Another reason may be that the HCV IRES and CrPV IRES are highly structured, and the rigid structures protect them from degradation; however, it is not the case for the EMCV IRES. The K164A/H165A mutation of SARS-CoV Nsp1 abolished its RNA cleavage function, indicating that binding to the 40S subunit is essential for RNA cleavage (Narayanan et al., 2008). Moreover, SARS-CoV Nsp1 with the R124A/K125A mutation still binds to 40S subunit, but it does not induce RNA cleavage (Lokugamage et al., 2012). It is thought that MERS-CoV Nsp1 induces host mRNA degradation in a different manner from that of SARS-CoV. A major difference between these two proteins is that SARS-CoV Nsp1 is a purely cytoplasmic protein whereas MERS-CoV Nsp1 localizes in both the cytoplasm and the nucleus (Prentice et al., 2004; Kamitani et al., 2006). It has been observed that MERS-CoV Nsp1 does not bind the 40S ribosomal subunit and only induces the degradation of the host mRNA of nuclear origin but not that of cytoplasmic origin. Moreover, MERS-CoV Nsp1 is reported to promote the assembly and budding of the virus particles (Nakagawa et al., 2018). MERS-CoV Nsp1 residues R146 and K147, the equivalent of SARS-CoV Nsp1 R124 and K125, also play key roles in Nsp1’s RNA cleavage function (Figure 2). Mutations of R146A/K147A in MERS-CoV Nsp1 abolish its ability to cleave host RNA (Lokugamage et al., 2012, 2015).

The mRNA cleavage induced by Nsp1 occurs through an indirect mechanism, as Nsp1 does not have a nuclease activity itself but can recruit exonucleases, such as Xrn1, to degrade host mRNA. Xrn1 is a highly conserved 5'–3' exoribonuclease involved in mRNA degradation in the cytoplasm (Nagarajan et al., 2013; Perez-Ortin et al., 2013; Labno et al., 2016). It has been observed that silencing Xrn1 in the cell abolishes the degradation effect of SARS-CoV Nsp1 and preserves the integrity of cellular mRNA (Gaglia et al., 2012). Other endonucleases that may be activated by Nsp1 have not yet been identified. Further studies are needed to understand the detailed mechanism by which coronavirus Nsp1 induces host mRNA cleavage.

In addition to the strategies mentioned above, Nsp1 may also inhibit the nuclear export of host mRNA to suppress the host protein expression. A recent study shows that SARS-CoV-2 Nsp1 interacts with the host mRNA export receptor heterodimer NXF1-NXT1 and prevents proper binding of NXF1 to mRNA export adaptors (Zhang et al., 2021). The NXF1-NXT1 complex is a general mRNA nuclear export factor that is conserved from yeast to human. The binding of SARS-CoV-2 Nsp1 to this complex restricts the export of host mRNAs from the nucleus and protein expression levels are subsequently downregulated (Zhang et al., 2021). This may explain why the Nsp1 K164A/H165A mutant fails to inhibit the expression of GFP which is transinfected directly into the cytoplasm, but decreases the expression of type I IFN even though the Nsp1 mutant does not have the ability to inhibit protein translation or induce host mRNA degradation (Narayanan et al., 2008). Moreover, SARS-CoV Nsp1 has been observed to associate with the nucleoporin Nup93 and displace it from the nuclear pore complex (NPC; Gomez et al., 2019). Similarly, SARS-CoV-2 Nsp1 can bind with NPC proteins, such as Nup358, Nup214, Nup153, and Nup62 (Zhang et al., 2021). These results indicate that Nsp1 has an additional capability of suppressing host protein synthesis by blocking mRNA transportation out of the nucleus.

## Nsp1 and Ribosome

The molecular mechanism of the interaction between the SARS-CoV-2 Nsp1 and the ribosome was recently elucidated by structural studies of the Nsp1-ribosomal complexes using cryogenic electron microscopy (cryo-EM) at the resolutions of 2.6–2.8Å (Schubert et al., 2020; Shi et al., 2020; Thoms et al., 2020; Yuan et al., 2020). These studies unambiguously identified that the C-terminal domain of Nsp1 (aa 145–180), which is disordered by itself (Almeida et al., 2007; Kumar et al., 2021b), folds into two helices upon binding to the mRNA entry channel of the 40S ribosomal subunit where it interacts with ribosomal proteins uS3, uS5, and eS30, as well as with helix h18 of the 18S rRNA (Figure 4; Schubert et al., 2020; Shi et al., 2020; Thoms et al., 2020; Yuan et al., 2020). Besides the C-terminal domain of Nsp1 bound in the mRNA channel, an additional weak globular density in the proximity of eS10 between the ribosomal protein uS3 and 18S rRNA helix h16 was also observed. The size of this density roughly matches the size of the N-terminal domain of SARS-CoV-2 Nsp1 (Figure 3; Clark et al., 2021; Semper et al., 2021). However, the local resolution of the cryo-EM maps in this region was low in all structures determined, which did not allow the modeling of the structure of the N-terminal domain of Nsp1 (Schubert et al., 2020; Thoms et al., 2020; Yuan et al., 2020). Moreover, as was noted by Schubert et al. (2020) this density may “correspond to unassigned ribosomal protein segments in the vicinity,” as the disordered C-terminal 65 amino acids of eS10 or the N-terminal 60 amino acids of uS5 could become better ordered in the context of Nsp1 binding.

Besides directly blocking the mRNA entry channel, a key feature of the C-terminal domain of Nsp1 is that it bridges the head and the body domains of the 40S ribosomal subunit, locking it in the “closed-state” conformation that is not compatible with the mRNA loading (Lomakin and Steitz, 2013; Yuan et al., 2020). Negatively charged amino acid residues D152, E155, and E159 of the C-terminal domain of SARS-CoV-2 Nsp1 interact with the positively charged residues R117, R116, R143, and K148 of uS3 of the 40S head. Furthermore, a large hydrophobic patch of the C-terminal domain of SARS-CoV-2 Nsp1 (F157, W161, L173, and L177) interacts with a hydrophobic surface on uS5 (V106, I109, P111, T122, F124, V147, and I151) of the 40S body. Finally, K164 and H165 of SARS-CoV-2 Nsp1 (the KH motif) establish stacking and backbone interactions with U607, G625, and U630 of the rRNA helix h18. In addition, two conserved arginine residues R171 and R175 of the C-terminal domain of SARS-CoV-2 Nsp1 interact with the phosphate backbone of h18. These extensive interactions result in the plugging of the mRNA entry channel by the C-terminal domain of Nsp1, physically preventing the loading and accommodation of the mRNA.

The structures of Nsp1-inhibited translation complexes with various initiation factors were also determined by cryo-EM (Schubert et al., 2020; Thoms et al., 2020). To this end, the HEK293 derived cell lysates were supplemented with Nsp1 and ribosomal complexes were purified either by affinity chromatography (Thoms et al., 2020) or by sucrose density gradient centrifugation (Schubert et al., 2020). The structural analysis revealed a range of complexes, including binary Nsp1-40S complexes, pre-40S-like complexes with ribosome biogenesis factor TSR1, 43S pre-initiation complexes (PIC) with eIF3 and different compositions of other initiation factors (eIF1, eIF1A, eIF2-tRNA_i_^Met^-GTP). Notably, all observed complexes did not have mRNA bound and the mode of the Nsp1-40S subunit interaction was identical. Altogether, these results are in aggrievement with the earlier proposal that SARS-CoV Nsp1 does not inhibit PIC formation, but rather suppresses 60S subunit joining to form the 80S ribosome (Kamitani et al., 2009). Interestingly, aforementioned structural analysis also revealed Nsp1-bound 80S complexes, which may seem to contradict the previous conclusion. However, in all cases, Nsp1 was bound to a translationally inactive 80S ribosome that does not possess mRNA (Schubert et al., 2020; Thoms et al., 2020). “Empty 80S ribosomes” without mRNA do not represent a usual, physiological state during protein synthesis. These nonproductive 80S ribosomes have the binding site for Nsp1 unoccupied and exposed, which then allow Nsp1 to bind.

Based on the location of Nsp1 on the 40S ribosomal subunit, it was hypothesized that Nsp1 may also interact/compete with the j subunit of the multi-subunit initiation factor eIF3 (eIF3j), which is involved in mRNA loading and binds to the 40S subunit in approximately the same region (Fraser et al., 2004, 2007; Aylett et al., 2015; Hershey, 2015; Sharifulin et al., 2016; Cate, 2017; Babaylova et al., 2019). The binding site of eIF3j to the 40S subunit, located in the mRNA channel, extends from the decoding center to the mRNA entry region (Fraser et al., 2007; Aylett et al., 2015; Hershey, 2015). It has been shown biochemically that Nsp1 competes with eIF3j for the binding to the 40S subunit, which likely weakens the binding of the eIF3 to the 40S subunit by disrupting uS3–eIF3j interaction (Yuan et al., 2020). Recently, studies including single-molecule fluorescence assay provided additional data to understand the function of the SARS-CoV-2 Nsp1 on the ribosome (Lapointe et al., 2021). Using a cell-free *in vitro* translation assay, Lapointe et al. (2021) showed that Nsp1 inhibits protein synthesis by specific, high-affinity interaction with the 40S subunit, independently from mRNA degradation. The single-molecule assay allowed to monitor real-time association of Nsp1 with the 40S ribosomal complexes and demonstrated that eIF3j competes with Nsp1 for the 40S subunit binding, consistent with the aforementioned results. Interestingly, this study also showed that eIF1 allosterically increases the Nsp1 affinity to the 40S subunit, likely by changing the conformation of the mRNA entry channel.

## Nsp1 and 5’-Utr

In the coronavirus lifecycle, the 5'-UTR of the viral genome plays a key role in the viral evasion of translation inhibition induced by Nsp1. The 5'-UTR region confers resistance to the RNA cleavage and translation inhibition induced by Nsp1. The 5'-UTR region is well structured, and the secondary structures in this region are very conserved in coronaviruses. There are five stem loops (SL1–SL5) in this region, with the start codon AUG embedded in the SL5 (Rangan et al., 2020; Miao et al., 2021; Sun et al., 2021). In SARS-CoV and SARS-CoV-2, SL1 alone is able to overcome the translation inhibition induced by the Nsp1, while other stem loops are not found to be important (Tanaka et al., 2012; Tidu et al., 2021). In SARS-CoV-2, swapping the locations of SL1 and SL2 ablates this protection. Additionally, the insertion of five extra nucleotides upstream of SL1 also abolishes the evasion of Nsp1 inhibition, indicating that strict steric restraints are needed in the progress (Banerjee et al., 2020). RNA sequencing studies show that all the SARS-CoV-2-encoded subgenomic RNAs that are translated into viral proteins contain a common 50-nt leader sequence. The leader sequence contains the SL1 region and protects the viral mRNAs from the translation inhibition induced by Nsp1 (Banerjee et al., 2020; Kim et al., 2020). A similar leader sequence is also found in the SARS-CoV-encoded subgenomic RNAs (Sola et al., 2015; Snijder et al., 2020).

It is interesting that the interaction of SL1 and Nsp1 only occurs when Nsp1 binds to the ribosome (Tidu et al., 2021). The SARS-CoV-2 5'-UTR itself cannot bind to the 40S or 60S subunit directly by itself, but it is able to promote the assembly of the pre-initiation complexes even in the presence of Nsp1 (Tidu et al., 2021). The viral RNA of coronaviruses has a 7-methylguanosine (m7G) cap on the 5' terminus, similar to that of human mRNA. The capped RNA recruits translation initiation factors to regulate protein expression. Initiation factors such as eIF4F and eIF4A are probably involved in the pre-initiation assembly for the viral RNA (Nakagawa et al., 2016). Computation modeling of the structure of SARS-CoV-2 Nsp1 and SL1 complex indicates that Nsp1 may interact with the SL1 in a clap-like fashion with a long antiparallel beta-sheet binding to the SL1 RNA helix directly (Vankadari et al., 2020). However, the critical role of 40S ribosomal subunit in the Nsp1-SL1 interaction remains unclear. In SARS-CoV and SARS-CoV-2, residues R124/K125 of Nsp1 that are highly conserved in beta-CoV, play key roles in viral evasion of translation inhibition. The R124A/K125A mutation of Nsp1 ablated the 5'-UTR-mediated evasion of translation of viral genes (Tanaka et al., 2012). Inhibitors targeting Nsp1 can likely abolish host translation inhibition or block the binding of viral 5'-UTR RNA to decrease the viral pathogenicity and replication. Considering that the molecular structure of the core domain of Nsp1 from different coronaviruses is conserved, this protein may be a suitable target for the development of broad-spectrum drugs.

## Nsp1 and Vaccine

Vaccination is one of the most effective methods against infectious pathogens. There are several different types of vaccines, including the inactivated vaccine, live attenuated vaccine, subunit vaccine, and the mRNA vaccine that has been successfully employed in the COVID19 pandemic. Nsp1, as a major pathogenicity factor of the coronaviruses, is a promising target gene for the live attenuated vaccines, which consist of replication-competent viruses that induce broad cellular and humoral immune responses without causing disease. Live-attenuated vaccines usually induce long-lasting immune responses and may provide life-time protection against the specific pathogen. Studies have been performed to develop live-attenuated vaccines against the coronaviruses (Zust et al., 2007a; Lei et al., 2014; Jimenez-Guardeno et al., 2015).

Mouse hepatitis virus (MHV) belongs to the beta-CoV genus and causes a wide range of disease symptoms in mice. MHV is widely used as a model in different viral studies, including vaccine tests. MHV with a 99-nt or 27-nt region deletions of the Nsp1 gene were found to be attenuated, restricted replicating in secondary lymphoid organs and protected mice from the infection of homologous and heterologous viruses (Zust et al., 2007b; Lei et al., 2014). Similar tests were also performed on SARS-CoV, where small region deletions in the C-terminal domain of Nsp1 (121–129 or 154–165) resulted in virulence attenuation, restored the IFN responses, and did not cause significant changes in the mouse lungs. The mice that were treated with the Nsp1 C-terminal region deletions survived the challenge with the virulent mouse-adapted SARS-CoV virus, indicating that Nsp1 variants with C-terminal region deletions are potential vaccine candidates (Jimenez-Guardeno et al., 2015). In addition, Nsp1 mutations were combined with other viral protein mutations, such as the envelope (E) protein, to increase both vaccine safety and stability. A SARS-CoV mutant with Nsp1 C-terminal region deletions and E protein region deletion was genetically stable after the virus passaged 10 times in the Vero E6 cells, was attenuated and induced cellular defense against the wild-type virus infection (Jimenez-Guardeno et al., 2015). These results suggest that Nsp1 is a promising target for the development of live attenuated vaccines.

## Conclusion and Future Perspectives

Coronavirus Nsp1, among the first viral proteins to be expressed after virus entering into the host cells, is a major viral pathogenicity factor of the alpha- and beta-CoVs. Nsp1 alters the host protein expression globally and inhibits the host immune responses by interfering with multiple steps of the immune response pathways, especially the Type I interferon pathway. Nsp1 inhibits host protein expression in multiple manners, including decreasing the nuclear export of host mRNA, inducing the host mRNA degradation and suppressing the host protein translation directly by binding to the ribosome. In the case of SARS-CoV and SARS-CoV-2, the C-terminal domain of Nsp1 directly inserts into the mRNA entry channel on the 40S ribosomal subunit and blocks the host mRNA loading for the translation initiation. However, viral RNA is able to escape the cleavage and translation inhibition *via* its 5'-UTR region. Among the conserved secondary structures in the 5'-UTR region, SL1 plays key roles in this evasion process, and studies show that strict steric restraints are necessary. Although the N-terminal core domains of Nsp1 proteins from different coronaviruses contain very similar 3D structures, Nsp1 proteins act through differing manners to inhibit the host protein expression. Many mechanisms surrounding Nsp1 are still unknown, such as how the core domain of Nsp1 acts in translation inhibition, how the host RNA is degraded, and how the viral genome escapes translation inhibition or RNA cleavage. Coronavirus Nsp1 is a suitable target for the development of live attenuated vaccines with deletions in key regions of Nsp1. Ongoing and future studies about the coronavirus Nsp1 will be valuable in defending against coronavirus infections in the current and potential future pandemics.

## Author Contributions

SY, SB, and IL conceived, designed, and wrote the manuscript. SY and SB prepared the figures. YX oversaw and edited the manuscript. All authors contributed to the article and approved the submitted version.

## Conflict of Interest

The authors declare that the research was conducted in the absence of any commercial or financial relationships that could be construed as a potential conflict of interest.

## Funding

This research was supported by Yale University Discretionary Funds to YX.

## Publisher’s Note

All claims expressed in this article are solely those of the authors and do not necessarily represent those of their affiliated organizations, or those of the publisher, the editors and the reviewers. Any product that may be evaluated in this article, or claim that may be made by its manufacturer, is not guaranteed or endorsed by the publisher.
